# 

**Published:** 1871-03

**Authors:** 


### BOOK NOTICES.

"Dr. Deem's Sermons," a medium for the circulation of the Gospel, as preached from the pulpit of the Church of the Strangers. Mailed post-paid to subscribers every week for $2.50 a year. Address LITTLE, RENNIE & Co., 645 Broadway, New York.

"The Young Pioneers," another of the Frontier Series, illustrated, by OLIVER OPTIC. Published by LEE & SHEPARD, Boston, and LEE, SHEPARD & DILLINGHAM, New York. Also by same, "Sophie Schwartz," from the Swedish.

"London Lancet," republished monthly at 52 John Street, New York, by WM. C. HERALD, $5 a year, being a standard record of medical and surgical progress throughout the world.

"Boston Medical and Surgical Journal," monthly, $4 a year. Unnoticed books are such as are sent to us, which are either unworthy of notice or are unfit for it, even by title. Books of that kind, even from respectable publishers, are becoming more numerous, and, under attractive titles and splendid binding, find their filthy way into households. Religious publications would do well to read all medical or physiological books sent to them before giving them any notice whatever. Not a few are profane and infidel as well as obscene; to notice such as they merit, would stimulate curiosity, and give them a wider circulation.

"The North American Journal of Homeopathy." Published quarterly at $4 a year by BOERICKE & TAFEL, 145 Grand Street, New York; San Francisco, 234 Sutten Street; London and Manchester, by HENRY TURNER & Co. No. 2, of the second series, contains several optical and surgical articles of great practical value. Doctors F. W. HUNT and S. LIEIENTHAL, editors.

Some pertinent and judicious remarks are made by the editors in their notices of a class of books of a particular kind, earnestly wishing that there were more men possessing the fearlessness of Rev. Dr. Dix of Trinity Church, in denouncing certain social sins. The editors would have done better to have gone a little deeper down, or higher up, we don't pretend to say which. Wonder if the reader wants us to stop here, or go a little further, and be a little plainer. Without further explanation or apology, we mean to say that the Roman Catholic Church teaches in her devotional books, in the plainest manner possible, that it is nothing short of one of the most heinous of all sins, being an execrable and cowardly murder to destroy the life of one begotten; as much a murder if done the instant after conception as if done before birth, or after; a foul, Heaven-daring murder all the way through. This is the only true doctrine. We would have saved ourselves the trouble of answering a good many letters in the past, and trust we shall save time in the future, by making this announcement as our belief, that whatever a married person does with a view to prevent having children is quite as great a crime against the race, in the sight of God, as cutting a born infant's throat before it is a day old. So far from doing anything to prevent having children, married persons ought to have all the children they can in honorable marriage; life will be longer, happier, and better, by this course, in the long run. Divinity intended the perpetuation of the race, and no man need pray

"have me excused," unless, by no fault of his own, he is no man at all; then he is a nobody, a cumberer of the ground, and the sooner he evaporates, the better. Let every man and woman in these matters stand in their own lot, and meet all life's responsibilities bravely, honorably, without shirking or without trickery. Such only are the true soldiers of the Mighty One. The Roman Catholic doctrine is the purest, the truest, and the best. Infanticide, fœticide even, is murder; dastardly, Heaven-defying murder. And let it be further understood that the effects of the efforts to prevent having children, whether before birth or impregnation, are corrupting to the mind and destructive of physical health and power in all cases, and inevitably so.

Dr. Hall's Publications. — John P. Sunderland, Esq., 40 Broadway, Room 44, New York, has opened an office for the sale of all the Editor's books, for receiving subscriptions, and obtaining advertisements, as well as for receiving subscriptions to "The Guide Board" to health, peace, and competence, or the road to a happy old age, by W. W. Hall, M. D., Editor of Hall's Journal of Health. This book is published by subscription only, by the enterprise of D. E. Fisk & Co., Springfield, Mass. It cannot be obtained at any bookstore. It is a royal octavo of 752 pages; both type and paper are very fine; the page is clear, open, and roomy, and is easily read. We have never read the book, since its publication, nor do we expect to do so; but taking the word of the publishers, it certainly treats of a good many interesting subjects.

It shows that food is the *best* physic.
It shows when and how to eat fruit.
It shows the farmer how and where to build houses and barns.
It shows the farmer what food is best for his cattle.
It shows how wives are *overtaxed.*
It shows how to live.
It shows how to grow old happily.
It shows how to die.
It shows how *every family* can save many times its cost every year.
It is good for each day and for all times.
It is free from medical terms, and so plain that all can understand it.
It exposes all humbugs.
It condemns quackery, and opens the eyes of those who *patronize* quacks, etc., etc.

All readers are advised to call at once, and subscribe for the book, in various styles, from four dollars up to seven, on John P. Sunderland, Esq., at No. 40 Broadway, New York, Room No. 44.

Cod Liver Oil is extensively advised by educated physicians for the cure of various diseased conditions of the human system. For all the purposes to which it is adapted, "Guffroy's Dragees" are the most desirable and efficient of all its varied preparations, because all the medicinal elements are retained, while they can be taken without any perception of its peculiarly disagreeable taste.

"John Swig," by Edward Casswell. Also "The Temperance Alphabet," with original designs, by same, published by J. W. Stearnes, publishing agent of the National Temperance Society and Publication House, 172 William Street, New York. We see noticed as just published "Laws of Fermentation," and "The Wines of the Ancients," by Rev William Patten, D. D. of New Haven.

"Insanity of Women," by H. R. Storer, M. D., L. L. B. Boston: & Lee Shepard, publishers. The book embodies a large amount of suggestive information; but the professional reader will be disappointed in it, while the common reader will be annoyed at finding so many pages of a purely personal controversial character, with the opinions of Tom, Dick, and Harry, on various points. The reading public wants facts and legitimate deductions, and not what the great Mogul or anybody else said or thought a thousand years ago. Dr. Storer has written several useful volumes; this on the Causation, Course, and Treatment of Reflex Insanity in Women is one of the least of these; a good many women are directly insane, with no "reflex" nonsense about it; for example, women doctors, women's rights folks, women's wrongs, and all that.

"Children's Week;" being seven stories for seven days, by R. W. Raymond, published by J. B. Ford & Co., 39 Park Row, New York, elegantly bound and beautifully illustrated for the little ones; every one of whom will be delighted with it.

"Minnesota as it is in 1871," by J. W. McLung, of St. Paul, containing a large amount of valuable and reliable information, just such as is needed by emigrants, invalids, tourists, capitalists, and business-men; with official statistics of population, extent, wealth, etc. etc., of each town and county, agricultural products, etc., etc.

Lee & Shepard, Boston, and Lee, Shepard & Dillingham, New York, have published for 1871, "The Tone Masters," "Mozart," "Mendelssohn," "Handel and Haydan," "Bach and Beethoven." Also, "Lost in The Fog," by James De Mille, a rousing story of the sea, illustrated.

"Every Day," by the author of "Katherine Morris," "Striving and Gaining," etc. by Noyes, Holmes & Co., Boston, Mass., 117 Washington Street; being twenty-eight chapters on as many interesting subjects, practical and suggestive, such as Looking Back, Foreshadowing, Tarlatan against Silk, First Fruits, Weary Hours, What the World said, Cross Purposes, etc., all conveyed in a beautiful story; bound elegantly in green and gold.

Hurd & Houghton have published "Sam Shirk, a Tale of the Woods of Maine," by George H. Devereux, being a record of ten years of the early manhood of the author amid the scenes and incidents described; a true and faithful delineation of the matters introduced. Nowhere in the wide world can a New Englander read this book without being carried back in imagination to the loved scenes and associations of boyhood, and be left all the better for it.

"Overland through Asia," being pictures of Siberian, Chinese, and Tartar life, in travels and adventures in Kamtschatka, Siberia, China, Mongolia, Chinese Tartary, and European Russia, with a description of the Amoor River and the shores of the Frozen Ocean, with an appropriate map and two hundred illustrations, (8vo, 608 pages,) by Thomas W. Knox, author of "Camp Fire and Cotton Field;" by the American Publishing Co., at Hartford, Conn. It cannot be had at the book-stores, as it is a subscription book; persons wanting it can address the Company at Hartford, Conn., or F. G. Gilman & Co., Chicago, Ill.; Nettleton & Co., Cincinnati, Ohio; H. H. Bancroft & Co., San Francisco; and an Agent will call on them. This is a new book, giving as it does a vast amount of information in reference to people and lands seldom visited, imparted in a style so graphic and life-like, so faithful to truth and fact, that it will be a satisfying feast to every intelligent reader.

"Bright's Disease," or the structure, functions, and diseases of the Kidneys, by EDWARD H. DIXON, M. D., published by J. S. REDFIELD, 140 Fulton Street, New York, and sent postpaid for thirty cents; this is a valuable tract, adapted to popular comprehension by an educated surgeon, who thoroughly understands his subject.

"Suburban Sketches," 234 pages, 12mo, published in elegant style by HURD & HOUGHTON, 13 Astor Place, New York. The author is W. D. HOWELLS, whose descriptions of "Venetian Life," and whose "Italian Journeyings," were so favorably received by the public not a great while ago. The "Sketches" are pictures of New England Life as seen near Boston, of Door-step Acquaintances, of Pedestrian Tours, Boston Horse Cars, Days of Pleasure, the Romance of Sea Life, of Old Scenes, and "Jubilee Days," and Flittings by. Every appreciative reader in New England or out of it will find a feast of pleasure and instruction in the perusal of the pages of HOWELLS' "Suburban Sketches."

A public service has been done by the same enterprising house in publishing in elegant style the Poems of LUCRETIA MARIA DAVIDSON, with illustrations by F. O. C. DARLEY, edited by M. OLIVER DAVIDSON. Some of our readers will remember the hearty reception given an age ago to the writings of the "Sisters Davidson;" and the "Poems" now published will be read with "pleasant memories" of the "light of other days."

"Flint's Practice of Medicine," published by HENRY C. LEE, Philadelphia, 1002 pages, 8vo, third edition, thoroughly revised by AUSTIN FLINT, M. D., Professor of the Principles and Practice of Medicine in the Bellevue Hospital, Medical College, New York. This volume is designed for the use of medical practitioners and medical students. This is a standard medical work, and has taken a high position in the estimation of the medical public, both at home and abroad, and is particularly valuable as being written by a gentleman whose extensive practice, whose large experience, whose accurate observation and acknowledged professional ability have admirably fitted him for presenting such a valuable publication to the medical profession.

"The Prophylactic Fluid," mentioned in one of the advertisements, is worthy of a place in every household for its safely purifying effects in all external applications. It is a good tooth-wash, preventing decay and purifying the breath, and is excellent for removing noisome odors from sinks, cellars, gutters, dirt heaps, etc.

"Laws of Fermentation and the Wines of the Ancients," by REV. WM. POTTER, D. D., published by the NATIONAL TEMPERANCE SOCIETY, 172 William Street, New York. This book will be considered an authority in the future, having been prepared with great care and conscientious fidelity by one of the best and most useful men of our time, who has in this manner done a lasting good for the friends of temperance in all climes.

The "American Tract Society," 150 Nassau Street, New York, has issued several beautiful, useful, and instructive volumes for the children who are so soon to become men and women, viz.: Grandmama's Trunkful of Stories; The Dance and the Martyr; Charity's Birth-day Text; The Nevers; Poems of Home Life; The Corner House, or Kindness Wins, —good and safe books all.

